# Incidental finding of cardiac hydatid cyst during autopsy

**DOI:** 10.4322/acr.2024.501

**Published:** 2024-06-21

**Authors:** Nishant Purbey, Amit Patil, Shreekant Bharti, Keshav Chandra, Shashank Ranjan

**Affiliations:** 1 All India Institute of Medical Sciences, Department of Forensic Medicine and Toxicology, Patna, Bihar, India; 2 All India Institute of Medical Sciences, Department of Pathology, Patna, Bihar, India

**Keywords:** Echinococcus, Electric Injuries, Parasitic diseases, Autopsy, Forensic Pathology

## Abstract

Hydatidosis or echinococcosis is an endemic parasitic disease caused by the ingestion of eggs of echinococcal species worldwide. In India, the annual incidence varies from 1 to 200 per one 100,000 hab., with the highest prevalence reported in the Indian states of Andhra Pradesh and Tamil Nadu. The dog is the definitive host, while humans, sheep, and cattle are intermediate hosts. The disease usually involves the liver and lungs, with the kidney and other organs rare involvement. Cardiac hydatidosis is still further rare, seen in 0.2% to 2% of the patients who remain asymptomatic until the development of its complications. Sudden deaths in cardiac echinococcosis are mostly attributed to cardiac arrhythmias, coronary artery diseases, valvular diseases, cardiomyopathies, pericarditis, and cardiac tamponade. We, herein, report a rare case of cardiac hydatid cyst incidentally found during the autopsy of a 26-year-old male who died due to electrical injuries. A single greyish-white cystic mass measuring 1.5cm X 1.2cm was detected on the left anterior ventricular wall 4 cm above the apex and was confirmed microscopically as a hydatid cyst. The cause of death was attributed to external injury.

## INTRODUCTION

Hydatid disease, also known as echinococcosis, is a parasitic infection caused by the larval stage of *Echinococcus granulosus* tapeworms. The disease is widespread worldwide but endemic in the Middle East, South America and Mediterranean countries.^1^ It is endemic in India, with annual incidence varying from 1 to 200 per 100,000 population,^2^ with the highest prevalence reported from Andhra Pradesh and Tamil Nadu due to of poor hygiene and cattle and sheep rearing population. It is transmitted by ingesting food, water, or soil contaminated with stool of infected cattle.^3-5^ The alimentary tract is the most frequent route via which*Echinococcus granulosus*is transmitted. Airborne bronchial venule penetration to the heart and systemic circulation is yet to be proven. It is also possible that parasites could bypass the portal filter and enter organs and tissues other than the liver and lungs through a lymphatic or venous shunt. Considering the muscle produces lactic acid, it is possible that*Echinococcus granulosus*eggs could hatch in soft tissues as well as the gastrointestinal tract in cases of involvement of the skeletal or cardiac muscles.^6^ For *Echinococcosis granulosus, the* dog is the definitive host; sheep, cattle, goats, and pigs are the usual intermediate hosts.^7^ Hydatid disease in humans most commonly occurs in the liver (55-70%) and lungs (18-35%), with rare involvement of kidneys and other organs.^8,9^ Cardiac involvement in echinococcosis is sporadic, usually asymptomatic until the development of complications.^9^ We report a rare case of cardiac hydatidosis that was incidentally found during a routine autopsy of a man who died of external injury.

## CASE REPORT

**Figure 1:**
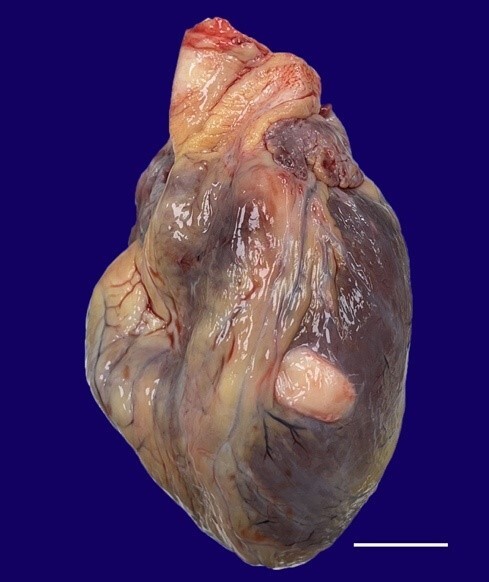
Gross view of heart with the cyst in the anterior ventricular wall (scale bar = 5cm).

A 26-year-old male was found collapsed on an air-filling machine while working after an electrical shock. The autopsy was conducted the next day. On external examination, the corpse was of a young adult male, average built, 161 cm in length. At autopsy, there was multi-visceral congestion without any internal hemorrhage. Internal organs were unremarkable except for the heart (weight 380 g, mean reference range 327 g), with a soft greyish-white nodule on the left ventricle anterolateral aspect measuring 1.5cm X 1.2cm and was 4 cm above the apex (Figure 1). Externally, the cystic swelling was fixed and extended into the left myocardial ventricle wall. The remaining heart examination, on sectioning, was unremarkable.

**Figure 2:**
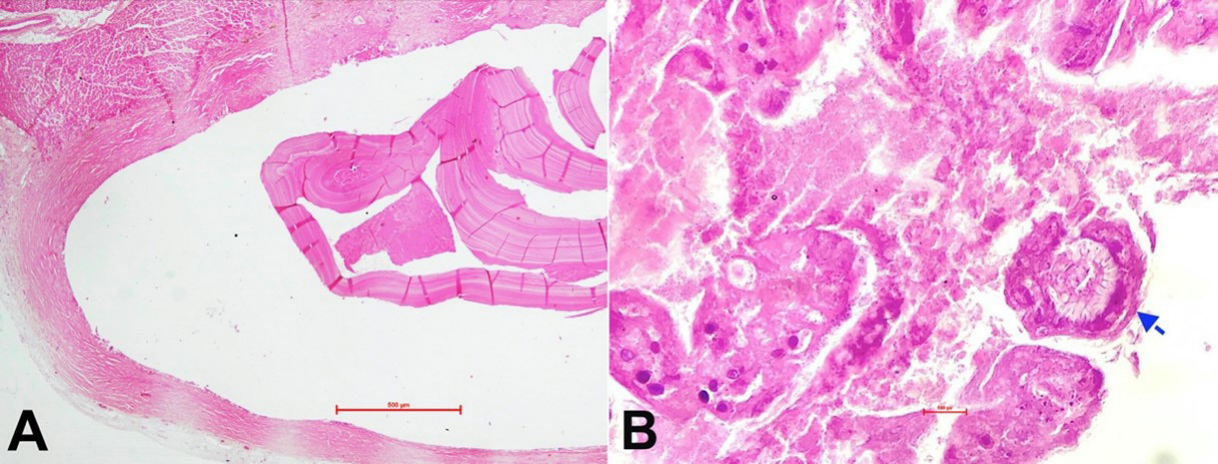
**A –** Superficial myocardium showing a cystic cavity with parasitic structure within the cyst (H&E, 10x); **B –** Hydatid cyst showing predominantly acellular wall and a protoscolex with refractile hooklets inside (Blue arrow) (H&E, 40x).

Microscopic examination using H&E staining of the paraffin sections showed a cyst overlying the myocardial surface (Figure 2A, 2B). The presence of a tortuous linear lamellate structure showing hyalinized amorphous eosinophilic wall and tiny brood capsules on one side of its surface was depicted. The cyst was formed and walled off by a thick fibrous capsule, thus confirming the diagnosis of a cardiac hydatid cyst. Histological findings of the ventricles and coronary arteries were unremarkable.

## DISCUSSION

Cardiac involvement by *Echinococcus granulosus* is seen in 0.2% to 2% of the patients who remain asymptomatic for a longer duration and can display nonspecific symptoms such as chest pain, cough, and palpitations.^10-13^ Existing 37 literature on cardiac hydatidosis suggests sample size varies from 2 to 62.^14^ The cysts usually grow slowly (1–5 cm per year) without causing symptoms, with probably only 10% of patients, especially those with large hydatid cysts, having clinical manifestations. The signs and symptoms of cardiac hydatid cysts are highly variable and directly related to the location and size of the cysts.^15,16^

Diagnosing cardiac hydatidosis in the early phase is difficult due to the long latency from exposure to the infection and the manifestation of the disease. Mostly, they are asymptomatic but can present clinically with chest pain, dyspnea, palpitations, arrhythmias, and AV nodal block. T wave inversion and premature ventricular beats are electrocardiographic findings.^17-19^

Chest radiograph findings are nonspecific except for the detection of cardiomegaly, which depends upon the size and site of the lesion.^20^ Echocardiography is the diagnostic method of choice for cardiac hydatidosis. It is an efficient, easy-to-perform, informative, and sensitive noninvasive technique to localize and detect cysts in living individuals. The most commonly used serology is the echinococcus indirect hemagglutination (EIHA) test and enzyme-linked immunosorbent assay (ELISA). However, a confirmatory diagnostic test for cardiac hydatidosis is the CT scan.^21^ Patients were managed surgically and pharmacologically, and their outcomes were good, so mortality was negligible. In the present case, the cyst was located on the anterior surface of the left ventricular free wall, one of the most common cardiac sites affected by the hydatid cyst owing to its thickness and rich blood supply, followed by the interventricular septum.^22,23^ The discovery of a hydatid cyst at autopsy requires confirmation by histopathology, an essential criterion standard for identifying the hydatid nature of the cyst. Microscopically, the wall of the hydatid cyst has 3 structural components: an outer acellular laminated membrane, the germinal membrane, and the protoscolices (Figure 2A, 2B). The hydatid cyst may be surrounded by a fibrous capsule or granulation tissue, including inflammatory infiltrate.^24^ In our case, histological examination confirmed the hydatid nature of the cyst in the heart.

Sudden deaths caused by hydatid diseases are rarely reported in the literature. In these, death is either attributed to the cystic rupture, embolization of hydatid material to the cerebral circulation or to the pulmonary artery, and anaphylaxis.^25-26^ However, in cases of cardiac hydatidosis, sudden deaths are mostly attributed to major causes such as cardiac arrhythmias, coronary artery diseases, valvular diseases, and cardiomyopathies.^27,28^

In the present case, though a cardiac hydatid cyst was confirmed as an incidental finding, the cause of death was attributed to an external cause.

## CONCLUSION

Cardiac echinococcosis is a rare disease that may remain asymptomatic until the development of complications and may be lethal in the absence of early diagnosis. In the present case, a rare incidental finding of a cardiac hydatid cyst was observed at autopsy. This was confirmed histologically. Cardiac hydatidosis can cause sudden death due to cardiac arrhythmias; however, that was not the cause of death in our case.

## References

[1] Gun E, Etit D, Buyuktalanci DO, Cakalagaoglu F (2017). Unusual locations of hydatid disease: a 10-year experience from a tertiary reference center in Western Turkey. Ann Diagn Pathol.

[2] Parija SC (2004). A textbook of medical parasitology.

[3] Reddy CR, Narasiah IL, Parvathi G, Rao MS (1968). Epidemiology of hydatid disease in Kurnnol. Indian J Med Res.

[4] Amir-Jahed AK, Fardin R, Farzad A, Bakshandeh K (1975). Clinical echinococcosis. Ann Surg.

[5] Mathur PN, Parihar S, Joshi CP, Kumawat JL (2016). Hydatid disease-still endemic in the southern region of state of Rajasthan, India: a clinical study carried out in tertiary care hospital. Int Surg J..

[6] Vecchio R, Vecchio V, Intagliata E (2020). Transmission ways of Echinococcus granulosus in rare muscular locations of hydatid disease. Ann Med Surg (Lond).

[7] Alexander J, Sharpe MH, Cotran RS, Kumar V, Robbins SI (2010). Robbins pathologic basis of disease.

[8] Büyük Y, Turan AA, Uzün I, Aybar Y, Cin O, Kurnaz G (2005). Non-ruptured hydatid cyst can lead to death by spread of cyst content into bloodstream: an autopsy case. Eur J Gastroenterol Hepatol.

[9] Farris AB, Petur Nielsen G, Kralin RL (2010). Diagnostic pathology of infectious diseases.

[10] Chadly A, Krimi S, Mghirbi T (2004). Cardiac hydatid cyst rupture as cause of death. Am J Forensic Med Pathol.

[11] Sahin I, Ozkaynak B, Ayca B, Okuyan E (2015). An uncommon localization of a giant hydatid cyst presenting with cardiac tamponade. Turk Kardiyol Dern Ars.

[12] Johnstone MT, Notariani M, Charlamb M (2000). Images in cardiovascular medicine: ventricular tachycardia as a complication of an intramyocardial echinococcal cyst. Circulation.

[13] Hosseini M, Hedjazi A, Bahrami R (2014). Sudden death due to anaphylactic shock in a patient with an intact hepatic hydatid cyst. Am J Forensic Med Pathol.

[14] Banisefid E, Baghernezhad K, Beheshti R (2023). Cardiac hydatid disease; a systematic review. BMC Infect Dis.

[15] Malamou-Mitsi V, Pappa L, Vougiouklakis T (2002). Sudden death due to an unrecognized cardiac hydatid cyst. J Forensic Sci.

[16] Ben-Hamda K, Maatouk F, Ben-Farhat M (2003). Eighteen year experience with echinococcosus of the heart: clinical and echocardiographic features in 14 patients. Int J Cardiol.

[17] Tuncer E, Tas SG, Mataraci I (2010). Surgical treatment of cardiac hydatid disease in 13 patients. Tex Heart Inst J.

[18] Di Bello R, Menendez H (1963). Intracardiac rupture of hydatid cysts of the heart. A study based on three personal observations and 101 cases in the world literature. Circulation.

[19] Seth HS, Mishra P, Khandekar JV, Raut C, Mohapatra CKR, Ammannaya GKK (2017). A concomitant intramyocardial and pulmonary hydatid cyst: a rare case report. Rev Bras Cir Cardiovasc.

[20] Demircan A, Keles A, Kahveci FO, Tulmac M, Ozsarac M (2010). Cardiac tamponade via a fistula to the pericardium from a hydatid cyst: case report and review of the literature. J Emerg Med.

[21] Oraha AY, Faqe DA, Kadoura M, Kakamad FH, Yaldo FF, Aziz SQ (2018). Cardiac hydatid cysts; presentation and management. A case series. Ann Med Surg (Lond).

[22] Mesrati MA, Mahjoub Y, Ben Abdejlil N (2020). Case Report: sudden death related to unrecognized cardiac hydatid cyst. F1000 Res.

[23] Parvizi R, Namdar H, Bilehjani E, Bayat A, Sheikhalizadeh MA (2013). Simultaneous operation of hydatid cyst of the heart and liver: a case report. J Cardiovasc Thorac Res.

[24] Bektas S, Erdogan NY, Sahin G, Kir G, Adas G (2016). Clinicopathological findings of hydatid cyst disease: a retrospective analysis. Ann Clin Pathol.

[25] Byard RW, Bourne AJ (1991). Cardiac echinococcosis with fatal intracerebral embolism. Arch Dis Child.

[26] Byard RW (2009). An analysis of possible mechanisms of unexpected death occurring in hydatid disease (echinococcosis). J Forensic Sci.

[27] Mahjoub Y, Boussaid M, Mesrati MA (2022). Hydatid disease, an uncommon etiology of death in forensic practice. Am J Forensic Med Pathol.

[28] Pakis I, Akyildiz EU, Karayel F (2006). Sudden death due to an unrecognized cardiac hydatid cyst: three medicolegal autopsy cases. J Forensic Sci.

